# Comment: Injuries that Hurt – A Comment to “Embitterment – Conception of a Potential Moderator to Dysfunctional and Aggressive Behaviour in Children and Adolescents”

**DOI:** 10.1007/s40653-024-00638-1

**Published:** 2024-04-24

**Authors:** David J. K. Hardecker, Julian Schmitz

**Affiliations:** Department of Clinical Child and Adolescent Psychology, Institute of Psychology, University of Leipzig, Leipzig, Germany

**Keywords:** Feeling hurt, Attachment injuries, Narcissistic injuries, PTSD, PTED, Humiliation, Embitterment

## Abstract

This comment discusses the article of Balder and Linden (2022), who debate the existence and role of embitterment in child and adolescent psychopathology. This topic appears highly relevant, but broader literature should be integrated when talking about embitterment. First, we question the conceptualization of embitterment as an emotion. We conceptualize it as a long-term emotion episode that stems from events involving severe and unresolved hurt feelings, also referred to as attachment injuries and narcissistic injuries. When overly intense, they might lead to PTED. In contrast to Balder and Linden (2022), we argue that specific psychotherapeutic treatments exist. Nonetheless, we join Balder and Linden (2022) in calling for more attention for embitterment and hurt feelings and their role in developmental psychopathology.

Balder and Linden (2022) present a valuable contribution to enhancing our understanding of the role of embitterment in developmental psychopathology, including early – potentially traumatic – reactions. They suggest that embitterment, understood as an emotion, can not only be seen in adults but also in children, and appear to have severe implications for children’s mental health. They point to a lack of empirical studies investigating this emotion, its consequences, and treatment options in clinical child psychology and psychiatry. We welcome this theoretical advance as posttraumatic embitterment disorder (PTED) frequently causes substantial suffering in adults, and its correct diagnosis allows for a better understanding and treatment (Linden & Arnold, 2021). Furthermore, the recent change in DSM-5 (American Psychiatric Association & Association, 2013) frees posttraumatic stress disorder (PTSD) from its strong association with fear and allows for other peri-traumatic emotions. Thus, when and where children and adolescents show (posttraumatic) embitterment appears equally relevant.

Here, we take up the author’s request for debating embitterment in children and adolescents. Therefore, we compare the concept of embitterment with related concepts and show at which points findings and treatment options have been previously discussed. First, we look at embitterment construed as an emotion and argue that it largely overlaps with the emotion term of feeling hurt but also with specific forms of disappointment, humiliation, and feeling wronged. Second, we look for long-term reactions to severe hurtful events, discuss related concepts -- attachment injuries, injuries to the ego, narcissistic injuries/wounds, traumatic invalidation -- and put them in the context of current concepts of posttraumatic stress disorders and compare it with posttraumatic embitterment disorder. Interestingly, the latter is a topic that the target article completely neglects. Finally, we point to existing treatment options such as emotion-focused therapy and attachment-based family therapy, that specifically address such wounds.

## Embitterment as an Emotion?

The authors start by discussing embitterment as an emotion. However, “embitterment,” in contrast to “feeling bitter,” as understood in everyday language, refers to a long-term emotion episode (Alexander, 1960), not a single emotion. Emotion episodes can be defined as a “continuous emotion sequence resulting from the more or less continuous transaction with one given event or issue” (Frijda, 2007). Such long-term emotional episodes consist of coping with the eliciting situations and might thus include different emotions (Frijda, 2007). These typically result from situations of injustice, humiliation, and betrayal in the case of embitterment (Balder & Linden, 2022). Following Znoj et al. (2016), such situations are appraised as including an agent who insults or humiliates one’s ego personally, while the situation is perceived as uncontrollable. These appraisals are thought to lead to aggression with the desire to reinstall justice and defend one’s self-worth. This appraisal profile resembles a particular emotion term, which is more frequently used when children and adolescents talk about their own emotions: *feeling hurt* or *hurt feelings* (Mills et al., 2002). Feeling hurt is also synonymous with feeling wronged, feeling disappointed by someone – also referred to as person-related disappointment (van Dijk & Zeelenberg, 2002) or social disappointment (Engelmann et al., 2017), and is largely identical to humiliation as an emotion (Hardecker, 2020). It also resembles basic forms of resentment (Roughley, 2018) and “feeling offended” (Poggi & D’Errico, 2018). Disappointment, humiliation, resentment and feeling wronged also appear prominent when discussing embitterment (Alexander, 1960; Linden & Arnold, 2021).

## Hurt Feelings, Sulking Behavior, and Embitterment

Hurt feelings can be defined as an emotion that consists of the appraisal of an unfair devaluation by another significant and often close person, low controllability, and the tendency to withdraw from the interaction (Hardecker, 2020). Most typically, children sulk when feeling hurt (Hardecker & Haun, 2020). Sulking behavior appears to signal this perceived devaluation and the threat to break up the relationship as indicated by typical utterances of sulking children (e.g., “You are unfair”, “Leave me alone”) (Hardecker et al., 2021). After verbally communicating, children often become silent, turn away, go away, cross their arms, lower their head, avoid eye contact, pout their lips, or narrow their eyebrows while sulking (Hardecker et al., 2021). Sulking might present itself as angry or non-angry sulking (Hardecker et al., 2022). Sulking as an expression of feeling hurt might thus have the social function of motivating the perpetrator to repair. Successful reparation might lead to restoring one’s self-esteem and feelings of justice. However, sulking is also associated with punishment, another strategy to restore justice. The authors, in contrast, do not describe specific embittered reactions. However, they give an example of a child lying in the snow to punish their mother, which more appropriately classifies as sulking behavior. We argue that hurt feelings denote a foundational emotional experience that concerns the same themes as embitterment, but which does not necessarily lead to aggression as suggested for embitterment. We propose to speak of embitterment if hurt feelings are not resolved and lead to inhibited anger and feelings of revenge. Events that have lead to severe hurt feelings we refer to as narcissistic injuries or attachment injuries. Furthermore, feeling hurt represents a particular normative emotion, while embitterment typically refers to persistent and intense long-term emotional episodes (Alexander, 1960) – a distinction crucial for discussing posttraumatic embitterment disorder.

What do we know about the general development of feeling hurt? Indeed, we agree that studies on the emotion family of hurt feelings are rare (For a recent review of feeling hurt and related aspects see (Hardecker & Haun, 2020). Furthermore, the same authors found in several descriptive studies that sulking behavior develops during the second and third year of life (Hardecker et al., 2022). In an experimental study, they found that 6-year-old children but not younger children systematically felt significantly more guilt as a response to sulking when compared to children who faced angry and sad children in an interaction paradigm (Hardecker et al., 2023). The argument by Balder and Linden (2022) that embitterment-like phenomena are present in children, thus, is fully supported when viewed from the more fundamental concept of feeling hurt.

## Hurt Feelings and PTED

Feeling hurt per se is a normative emotion occurring in all or almost all children and adolescents. Psychopathological relevance emerges only if experienced too often or intensely, leading to substantial suffering and impairment of individual and social functioning. Balder and Linden (2022) review evidence that indicates that intense emotional reactions are present in children – following sibling rivalry or bullying and discuss their relevancy for oppositional defiant disorder (F91.3, ICD-10). However, they do not discuss whether posttraumatic embitterment disorder is present in children. Given the severity of particular hurtful situations such as bullying, abuse, or criticism, instances of such encounters might indeed lead to trauma-like reactions. Such reactions based on severe hurt feelings or humiliation have been referred to as *narcissistic injuries*, *injuries to the ego*, and *attachment injuries* (Diamond, 2014; Levin, 1993) and need to be distinguished from normative hurt feelings – analog to sadness and grief. Balder and Linden (2022) neglect these crucial distinctions.

In support of the argument that overly intense hurt feelings might have traumatic consequences in childhood also, several studies showed that symptoms of PTSD did not necessarily depend on the experience of trauma-related fear (Roemer et al., 1998). Other peri-traumatic emotions such as anger, shame, and disgust experienced in extreme ways instead of fear predict PTSD symptoms in victims of violent crime, for instance (Andrews et al., 2000). Dalgleish and Power (2004) accordingly suggested that PTSD might best be described as involving emotion-specific components and emotion-non-specific components with the latter including symptoms of intrusion and avoidance and the former including a variety of emotions. Taking up this line of evidence, the fifth edition of the *Diagnostic and Statistical Manual for Mental Disorders* (American Psychiatric Association & Association, 2013) includes PTSD in the new section of Trauma and Stress-Related Disorders, thus separating the diagnosis from its former place within anxiety disorders. The discussion of hurt feelings or embitterment in the context of PTSD is thus well founded.

Table 1 shows an overview of independently formulated concepts of hurt-based PTSD and associated treatment options. We discuss them in the following.

PTED consists of two core criteria (Linden & Arnold, 2021, S. 74)“1. The patient responds with embitterment in direct relation to a negative life event which is experienced as injustice, humiliation, or breach of trust. 2. The patient suffers from recurrent intrusional thoughts concerning the event and reacts with distinct emotional arousal when reminded of what has happened.” However, following Znoj et al. (2016), potentially embittering life events can lead not only to embitterment but also to depression, aggression, or personal growth; it might thus be fruitful to speak of embitterment as one class of hurt-based PTSD.” In line with this view, an aggressive component is included only as an additional symptom of PTED, although it appears necessary in the construal of embitterment.

Based on attachment theory and clinical experience, Johnson et al. (2001) formulated the concept of *attachment injury*, defined as “a specific type of betrayal that is experienced in couple relationships. It is characterized as abandonment or a violation of trust (…) it concerns a specific incident in which one partner is inaccessible and unresponsive in the face of the other partner’s urgent need for the kind of support and caring that we expect of attachment figures” (p.149). Similar to the concept of embitterment disorder, it highlights violation of trust. When viewed from attachment-based family therapy, criticism, control, abuse, and neglect are named as additional situations that cause attachment injuries (Diamond, 2014) – situations that often cause hurt feelings and appear to be well described by their emotion profile (Hardecker, 2020).

Psychoanalytic authors have discussed the notion of *narcissistic wounds or injuries* since its beginnings (Freud, 1917). It that has been defined as “a wound to the core self accompanied by feelings of humiliation, shame and rage” which “lowers self-esteem and disrupts our sense of self” (Levin, 1993). Goldberg (1973) defined it as “injuries of a psychologic nature to one’s self or self-esteem”, argued that it would represent a distinct diagnostic category with high relevance for depression, and explicitly linked it to hurt feelings. Furthermore, psychoanalytic authors usually highlight the central role of narcissistic injuries for child development in general and for developmental psychopathology in particular (Levin, 1993).

In summary, we argue that future work should relate PTED to these other concepts and formulate a more integrative concept based on feeling hurt.

**Table 1:** Concepts of hurt-based PTSD and corresponding treatment

Author	Concept	Central Appraisal or Emotion	Components of PTSD	Treatment
Levin (1993**)**	**Narcissistic injury**	hurt, humiliation, shame, disappointment	not explicitly mentioned	Psychodynamic Psychotherapy
Johnson et al. (2001)	**Attachment injury**	hurt	“the injured partner may exhibit symptoms that are characteristic of posttraumatic stress disorder **(PTSD)**, such as reexperiencing, numbness, and hypervigilance” (p.149)	Emotion focussed couple therapy, Attachment based Family therapy
**Linden (** 2003 **)**	**Posttraumatic embitterment disorder**	humiliation, unfairness	intrusional thoughts, emotional arousal	Wisdom Therapy

## Psychotherapeutic Approaches

Balder and Linden (2022) point out that embitterment has not been considered in child and adolescent psychiatry and psychotherapy and that wisdom therapy might represent a potential treatment option already used in adult psychotherapy. If we look for the expression of “embitterment” this appears true. But as we argued throughout this comment, several linguistic expressions refer more or less to the same phenomenon: social rejection, rejection sensitivity, humiliation, narcissistic injuries, or narcissistic wounds refer to a family of phenomena and should thus be related to each other. Indeed, wisdom therapy which is a form of cognitive therapy that involves the development of various competencies associated with wisdom such as perspective taking (changing perspective, self-distancing), empathy, acceptance, and humor (Linden & Arnold, 2021) appears to be a promising approach.

However, other psychotherapeutic approaches that target these phenomena exist. For instance, one effective treatment program for externalizing problems in children addresses vicious parent-child interaction cycles (Döpfner et al., 2013) that are – supposedly – based on hurt feelings on both sides. Rational emotive behavioral therapy has been adapted for hurt feelings in adults by Dryden (2012). For traumatic reactions in general, other approaches exist that are already used for traumatic reactions in children (Cohen et al., 2006; Sierau et al., 2019). Furthermore, there is one approach that was specifically developed to treat adolescents using a family therapeutic and systemic approach: Attachment-based family therapy provides us with an emotion-focused and trauma-based procedure to resolve attachment injuries – specifically but not exclusively in depressed adolescents (Diamond, 2014). Its central part consists of facilitating corrective attachment episodes in which children or adolescents express their hurt feelings while parents respond empathically by providing comfort, acceptance, understanding, and apologies. It is a brief family therapy that has been shown to be highly effective (Diamond et al., 2016).

## Conclusion

Balder and Linden (2022) point to an extremely relevant topic. However, they neglect a wide range of literature that is based on different terminology but refers to the same or closely related phenomena. Future work should aim at an integrative concept of injury-based traumatic reactions, based on a detailed description of such events and specifying injurious and embittered reactions more precisely. On this foundation, the role of traumatic hurtful events in various childhood mental disorders can then be described. It also allows the range of existing treatment options that target and potentially resolve such traumatic memories to be empirically evaluated. However, linguistic awareness is essential to avoid “the same wine in different bottles” and to avoid overlooking the wealth of psychotherapeutic treatment options.
